# Effects of flavour variety on the intake and palatability of commercial feed in nursery pigs

**DOI:** 10.3389/fvets.2023.1218198

**Published:** 2023-08-30

**Authors:** Elizabeth Huenul, Laura Salazar, Daniela Frias, Milivoy Videka, Daniela Luna, Dominic M. Dwyer, Jaime Figueroa

**Affiliations:** ^1^Department of Animal and Food Science, Faculty of Veterinary Medicine, Autonomous University of Barcelona, Cerdanyola del Vallès, Spain; ^2^Animal Behaviour and Welfare, Animal and Veterinary Sciences Research Group, Scotland’s Rural College, Edinburgh, United Kingdom; ^3^Escuela de Ingeniería y recursos Naturales, Instituto Profesional DuocUC, Santiago, Chile; ^4^Departamento de Fomento de la Producción Animal, Facultad de Ciencias Veterinarias y Pecuarias, Universidad de Chile, Santiago, Chile; ^5^School of Psychology, College of Biomedical and Life Sciences, Cardiff University, Cardiff, United Kingdom; ^6^Instituto de Ciencias Agroalimentarias, Animales y Ambientales, Universidad de O’Higgins, San Fernando, Chile

**Keywords:** consumption pattern, feed intake, flavour variety, nursery pigs, sensory specific satiety

## Abstract

Sensory-specific satiety (SSS) could negatively affect pigs’ feed intake, even when diets satisfy their nutritional requirements. We evaluated the short-term effects of SSS on feed intake and palatability. Thirty-two nursery pigs (tested in pairs) were exposed to short-term feeding trials for 6 days. In Trial 1, animals received for 90 min over three consecutive days three feeders: with different flavours (VAR); the same flavour (MON); or a mixture of the three flavours (MIX) in a 3 × 3 Latin square design. In Trial 2, with the same animals and different flavours, the three feeders were delivered successively (1 feeder every 30 min). In Trial 1, there was a day-by-diet interaction (*F* 4,36 = 2.98; *p* = 0.032), where the VAR diet was least consumed on the first day but most consumed subsequently. In Trial 2 a triple interaction between diet, day and delivery order modified pig’s intake (*F* 12,15 = 3.33; *p* = 0.015), and consumption patterns (*F* 12,15 = 3.52; *p* = 0.012); where VAR diet presented the highest values in the last delivery order on the third experimental day. Flavour variety may decrease the effect of SSS, increasing feed intake and hedonic value in nursery pigs when there was a previous experience with those flavours.

## 1. Introduction

Pigs in a natural environment are opportunistic and omnivorous feeders that during most of their active time search and consume an extensive variety of foods (1). Their specialized oro-nasal system allows them to search above and below the ground for a wide range of foods including plants, seeds, tubers, insects, fruits, small mammals, and even reptiles in order to satisfy their nutritional needs (2, 3). In contrast, pigs raised in conventional farming do not have the opportunity to search for different food resources, although the pig industry offers a complete diet according to their specific nutritional requirements at their different productive stages (4). Depending on their local availability and price these diets include several ingredients and additives. Nevertheless, even though feeds may contain additives that contribute to increasing palatability, a mixed diet has the potential to create a unified flavour experience (5). Moreover, the organoleptic properties of feed differ little between and within production periods, which can generate problems of sensory-specific satiety (6).

Sensory-specific satiety (SSS) is a physiological phenomenon, associated with the decrease in the specific hedonic value of the sensory properties of food after being continuously exposed during a feeding episode, and which recovers after time (6, 7). As an example, if someone allowed us to consume only our favourite food for several days, the sensation of pleasure when eating that food would diminish with the increased exposure. Thereby, sensory-specific satiety would be expressed as a decrease in the pleasantness of taste and a reduction in consumption relative to other foods that differ in one or more sensory properties, even if they have the same nutritional composition (8, 9).

Animals typically need to eat a varied diet to obtain all their required nutrients (10) and food macronutrients are associated with different sensorial qualities (11). Therefore, the SSS is considered an adaptive mechanism, one that ensures animals search the environment to obtain different nutrients through a varied diet to fit their physiological needs (12). The role of SSS and the adverse effects of feed’s sensory monotony has been studied mainly in humans (7) and rats (13, 14), but also in other domestic animals like sheep (15), where the absence of sensory variety over days can lead animals to reduce their intake, thus affecting their performance and welfare (16). However, when the humans or animals have the opportunity to eat diets whose sensory properties have been varied, they start increasing their intake again, even during the same consumption episode (17–19). In addition, a feed environment with a wide sensory variety allows the animal to express their feed preferences and natural feeding behaviour, potentially having an important effect on animal welfare (20). Such improvements in the performance and welfare of animals are the desired outcomes in animal production, such as pig farming.

The positive effect of the dietary sensory variety has been little addressed in pigs. Recent experiments suggest that during the suckling period, creep feed with sensory variety or dietary variety increases feed intake and exploratory behaviour in piglets compared to a sensory monotonous diet. However, no effect of diet variety was found in the performance parameters of piglets where similar weights and weight gain were observed at weaning (21, 22). Nevertheless, the maternal presence, with the constant availability of milk and the marginal consumption of solid feed could mask positive results of sensory variety in animals at this production stage. Therefore, it is necessary to understand the effect of sensory variety on pig feeding behaviour in other production stages. The objective of the present study was to evaluate the short-term effect of specific-sensory satiety on the consumption and palatability of flavoured feed in nursery pigs.

## 2. Materials and methods

Experiments were conducted at the swine experimental facility of the Centro de Investigación, Innovación Tecnológica y Capacitación para la Industria Porcina Nacional (CICAP), belonging to the Pontificia Universidad Católica de Chile (PUC) in Santiago, Chile. All experimental procedures were approved by the Ethical Committee on Animal Experimentation of PUC (No. 190531007).

### 2.1. Animals and housing

A total of 32 castrated male and female nursery pigs (PIC Genetics), 42 days-old (13.2 ± 1.2 kg) at the start of experiments, served as subjects. After weaning at 28 days-old, animals were individually identified by using numbered plastic ear tags, weighed and randomly allocated in pairs to 16 nursery pens (1.80 m × 1.28 m × 0.7 m, fully slatted floor), maintaining similar weights between pens (*p* > 0.05). The nursery room temperature (29°C lowering 1°C per week) was controlled with a heater and automatically forced ventilation. Each pen had one feeder with three feeding spaces and an individual water supply. Pigs were *ad-libitum* fed with an unflavoured standard commercial diet according to their nutritional requirements (4) and they had constant access to fresh water throughout the experimental procedure (except for the removal of unflavoured food during the period 1 h before and after each experimental session). The commercial formulation of feed was confidential but based mainly on Maize (611 g/kg), soy bean products (168 g/kg), fish meal (80 g/kg), sweet milk whey (89 g/kg) and a complete premix with vitamins-aminoacids-minerals and other additives to enhance feed digestibility. Environmental enrichment was not added to the pens. Animals were tested in two trials of three consecutive days each between 10 AM–12 PM, and the two trials were separated by a rest week. During the second trial, the feeding behaviour of animals was recorded with 8 video cameras (IR exterior 1/3 Sony^®^ 700tvl cmos; SENKO S.A, Santiago, Chile) distributed every two pens in the ceiling of the nursery room. The videos were downloaded at the end of the experimental period and were analyzed by a trained observer. Behavioural observations were analyzed using the Behavioural Observation Research Interactive Software [BORIS, http://www.boris.unito.it/ (23)].

### 2.2. Experimental procedure

**Figure 1:**
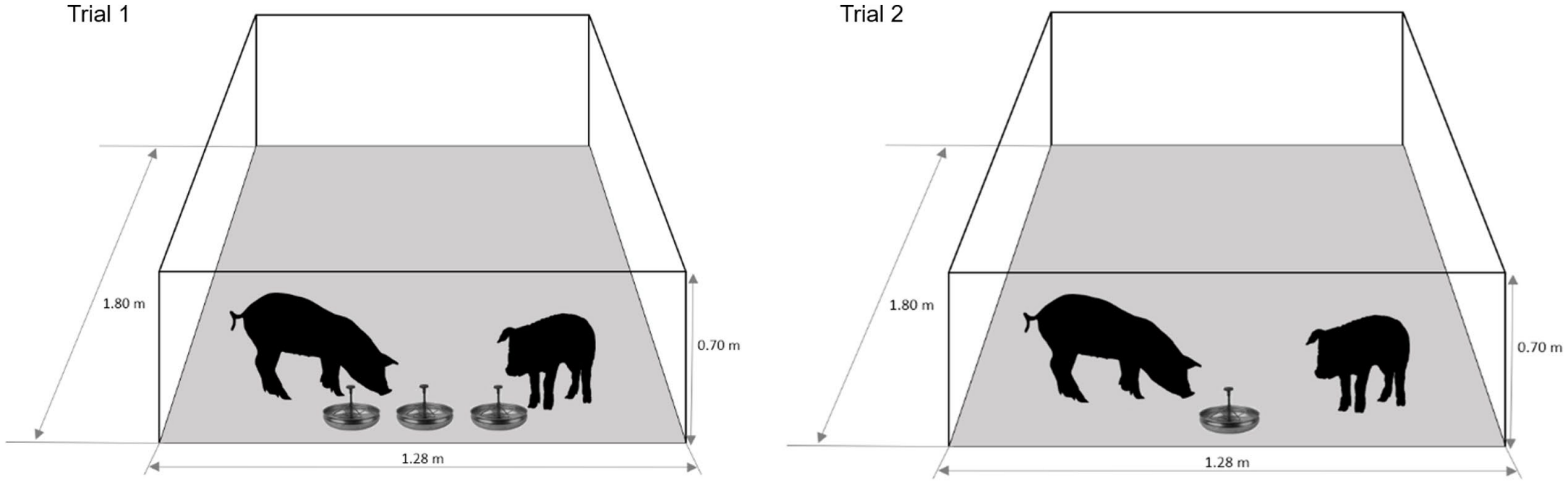
Schematic representation of the pen (front view) during Trial 1 and Trial 2 sessions.

**Figure 2:**
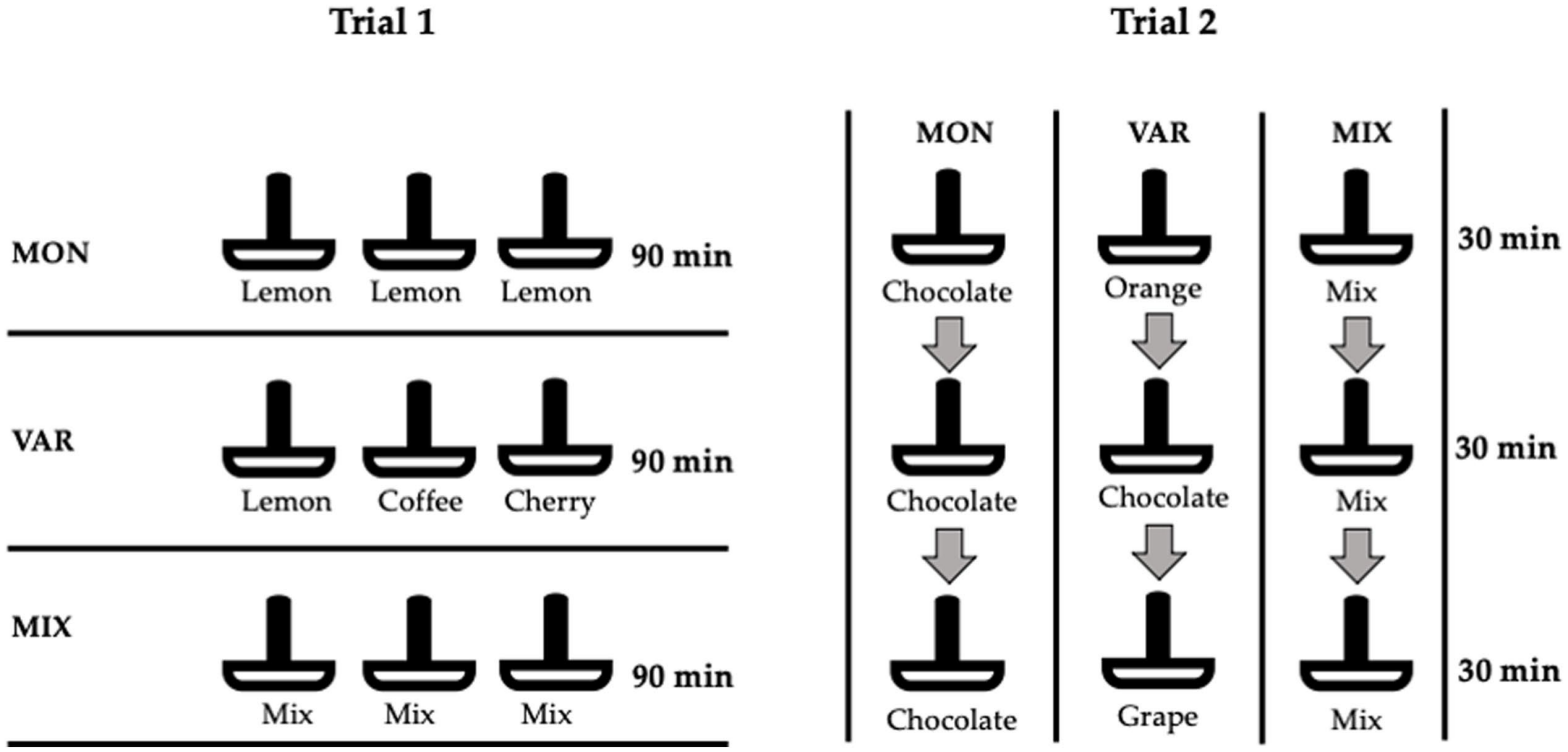
Schematic representation of monotonous (MON), varied (VAR), and mixed (MIX) diets delivered in both trials. In Trial 1, three pan-feeders were offered at the same time for 90 min. Lemon, coffee, and cherry flavours were used as added artificial flavours on feed. In Trial 2, one pan-feeder was offered every 30 min until completing 90 min. Orange, chocolate, and grape flavours were used.

Before the beginning of trials pigs were acclimated to housing and experimental conditions (28–41 days-old). Experimental schematic representation and procedures are summarized in Figures 1, 2, respectively. Two feeding trials were performed with the same animals. Each trial had a duration of 3 days, during which animals were exposed in the morning for 90 min to three pan-feeders with commercial feed that contained either: (1) different flavours (VAR); (2) the same flavour (MON); or (3) a mixture of the three flavours in each feeder (MIX). All animals experienced each of the three experimental conditions with the order counterbalanced in a 3 × 3 Latin square design. In Trial 1, the feeders were given simultaneously during the 90 min of the trial. Flavours added to the feed were lemon, coffee and cherry at 0.075% [(24); Floramatic^®^, Santiago, Chile], where lemon was used in the MON diet. A similar procedure was conducted in the second trial, but feeders were rotated every 30 min until the 90 min were completed and the flavours used were orange, chocolate and grape (Floramatic^®^ Santiago, Chile, 0.075%), where chocolate was used in the MON diet. Flavours used in both trials were selected based in previous unpublished trials and in the company recommendations, considering similar preferences and intake between them. Flavours used in Trial 1 and 2 were different to ensure that test flavours were novel at the start of each of Trial 1 and Trial 2. Their commercial unflavoured feed was removed 1 h before the start of each test and was returned to each pen 1 h after the end of the tests. Feed intake was measured by weighing the pan-feeders at the beginning and end of each test (spillage was not measured). During Trial 2 consumption time (time eating at the pan-feeder; CT) and approaches (number of times the pan-feeder was approached with a consumption result; A) were assessed from the video recordings by focal continuous sampling over the 90 min tests. Palatability was estimated through consumption patterns (CT/A) (25, 26), analogous to the licks/bout measure used in rats in lick cluster size analysis (27, 28).

### 2.3. Statistical analysis

Feed intake and consumption patterns were analyzed with ANOVA by using mixed linear models with the MIXED procedure of statistical package SAS^®^ (SAS Inst. Inc., Cary, NC, United States), considering the effect of the diet (MON, VAR or MIX), experimental day (1, 2 or 3), delivery order of the given diet during Trial 2 (first, second or third) and the interaction between variables. The pen was considered as a repeated measure in the mixed model. Before ANOVA analysis, the normality and homoscedasticity of the dataset were analyzed by using the UNIVARIATE procedure with the Shapiro–Wilk and O’Brien’s tests, respectively. The mean values are presented as least square means adjusted by Tukey. The experimental unit was the pen with results expressed as the average of both pigs’ data. Differences at *p* < 0.05 were considered statistically significant and differences at 0.05 ≤ *p* < 0.10 were considered a trend.

## 3. Results

### 3.1. Trial 1: simultaneous exposure to flavoured feed

**Figure 3:**
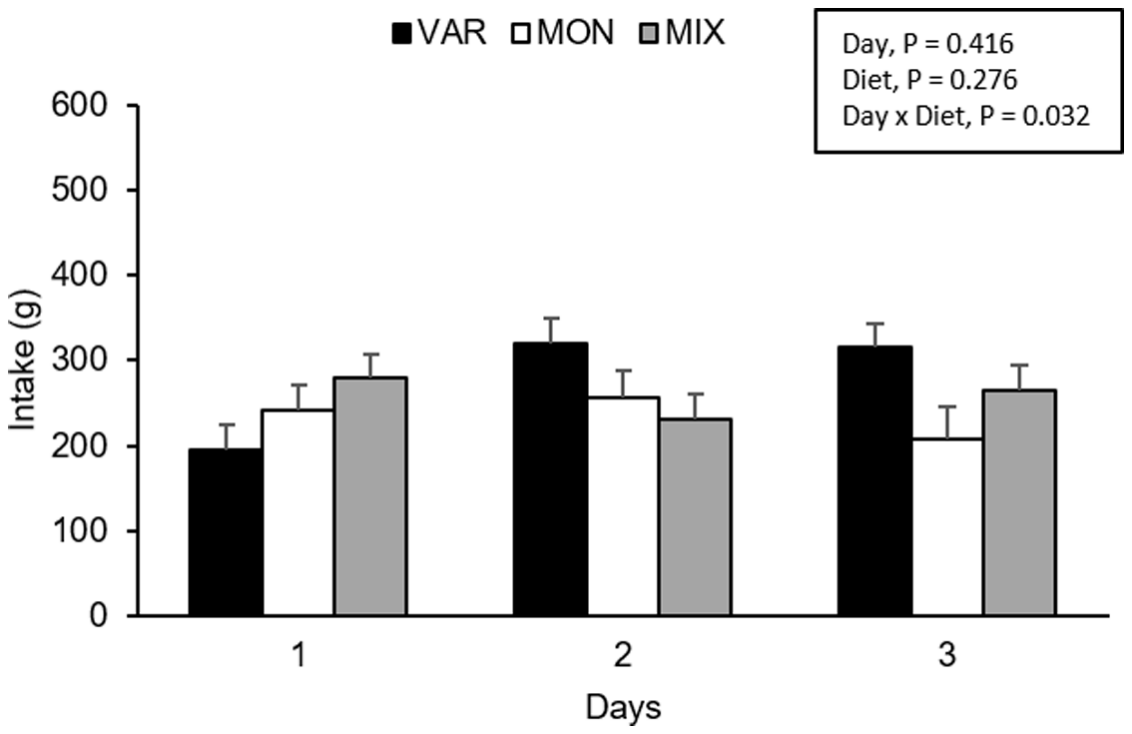
Total feed intake (mean ± SEM) of nursery pigs during a simultaneous exposure (90 min) of three feeders containing feed of different flavours (lemon, coffee, or cherry; VAR), with the same flavour (lemon; MON) and with a MIX of the three flavours (lemon + coffee + cherry). Results are expressed by pig and experimental day (1, 2, or 3).

No intake differences were observed in nursery pigs during Trial 1 according to the experimental day (*F* 2,36 = 0.90; *p* = 0.416) or diet (*F* 2,36 = 1.34; *p* = 0.276). However, a significant interaction between the experimental day and diet was found (*F* 4,36 = 2.98; *p* = 0.032), where the VAR diet showed the lowest intake on day one and the highest intake on days 2 and 3 compared to the other diets (Figure 3). By analysing separately, the effect of the day in each diet consumed, the intake of VAR diet varied between days (*F* 2,13 = 6.27; *p* = 0.012), presenting a significant increase in its intake between day 1 and 2 (*p* = 0.022) and from day 1 to 3 (*p* = 0.021) with no significant differences between day 2 and 3 (*p* = 0.990). Pigs equally consumed MIX diet (*F* 2,13 = 0.98; *p* = 0.403) or MON diets (*F* 2,10 = 0.36; *p* = 0.709) across days.

### 3.2. Trial 2: consecutive exposure to flavoured feed

**Figure 4:**
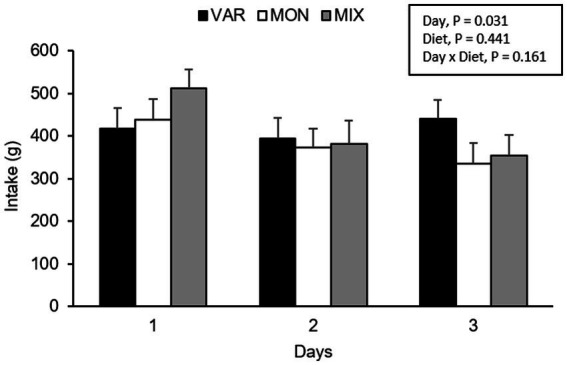
Total feed intake (mean ± SEM) of nursery pigs during a consecutive exposure of three feeders (for 30 min each) containing feed with different flavours (orange, chocolate, or grape; VAR), with the same flavour (chocolate; MON) and with a mixture of the three flavours (orange + chocolate + grape; MIX). Results are expressed by pig and experimental day (1, 2, or 3).

**Figure 5:**
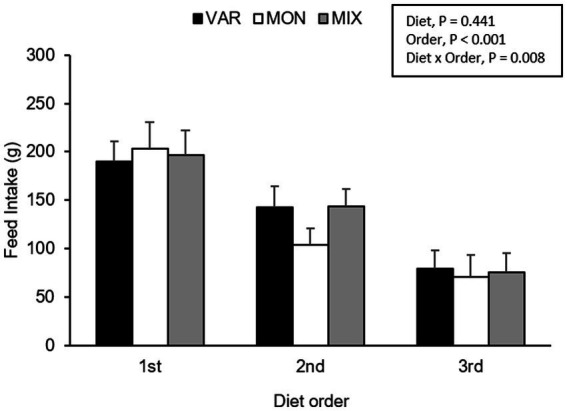
Feed intake (mean ± SEM) of nursery pigs during a consecutive exposure of three feeders (for 30 min each) containing feed with different flavours (orange, chocolate, or grape; VAR), with the same flavour (chocolate; MON) and with a mixture of the three flavours (orange + chocolate + grape; MIX). Results are expressed by pig and diet delivery order (1st, 2nd, or 3rd).

**Figure 6:**
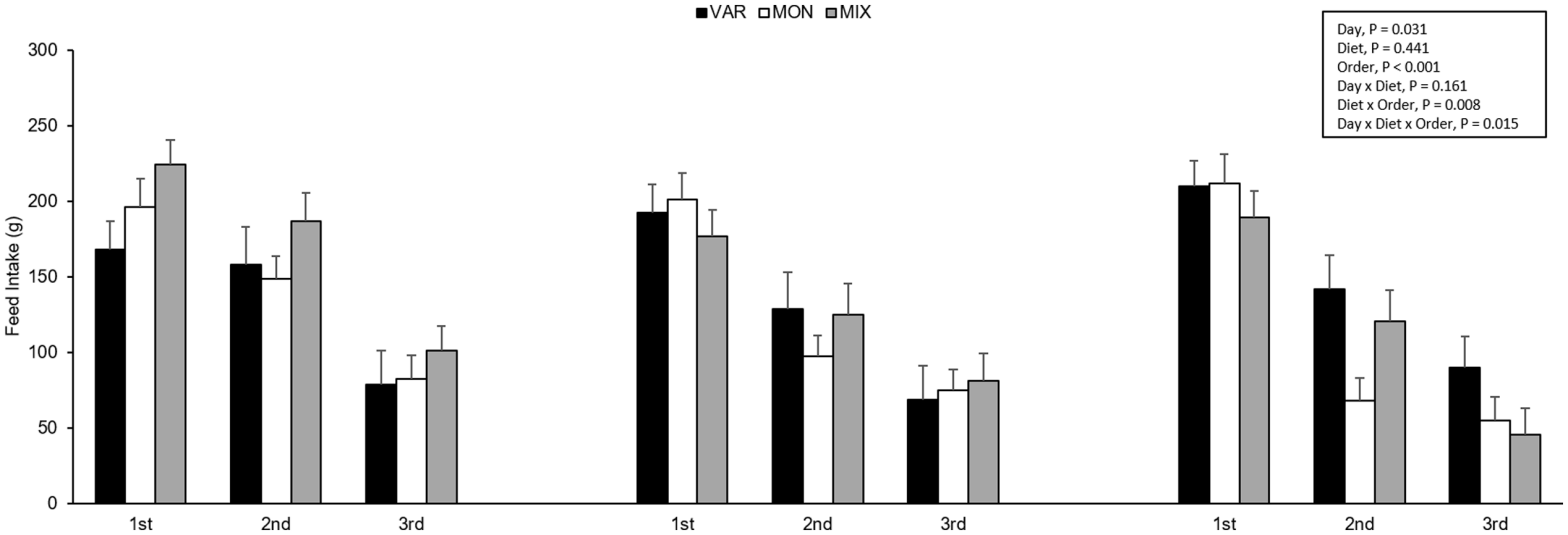
Feed intake (mean ± SEM) of nursery pigs during a consecutive exposure of three feeders (for 30 min each) containing feed with different flavours (orange, chocolate, or grape; VAR), with the same flavour (chocolate; MON) and with a mixture of the three flavours (orange + chocolate + grape; MIX). Results are expressed by pig, diet delivery order (1st, 2nd, or 3rd) and day (1, 2, or 3).

The experimental day and delivery order of the feed influenced pig’s intake, observing a lower consumption of the flavoured feed as the days go by (*F* 2,15 = 4.40; *p* = 0.031) and as the delivery order progresses (*F* 2,15 = 63.37; *p* < 0.001) respectively. No intake differences were observed in Trial 2 according to experimental diets (*F* 2,15 = 0.87; *p* = 0.441). The interaction between the diet and day is presented in Figure 4. Although it is observed that the VAR diet was the less consumed on day one but the highest on day 3, the interaction was not significant (*F* 4,15 = 1.91; *p* = 0.161). By analysing separately, the effect of the day in each diet consumed, pigs equally consumed the VAR diet (*F* 2,13 = 0.25; *p* = 0.779) across days. The intake of the MIX diet varied between days (*F* 2,12 = 6.23; *p* = 0.014), observing that animals decrease its consumption between days 1 and 2 (*p* = 0.041) and between days 1 and 3 (*p* = 0.021) with no differences between days 2 and 3 (*p* = 0.984). Finally, the intake of the MON diet did not significantly differ between days (*p* > 0.1). A significant interaction between diet and delivery order of feed was found (*F* 4,15 = 5.17; *p* = 0.008), observing that the MON diet presented the highest intake on the first exposure compared with the other treatments but the lowest intake on the last exposure (Figure 5). Finally, a triple interaction between diet, day and delivery order was observed (*F* 12,15 = 3.33; *p* = 0.015), where the variety diet presented the lowest intake during the last delivery on the first day, but the highest intake during the last delivery on the last experimental day (Figure 6): that is, the decrease in intake across the session was lowest in the VAR condition once all flavours were familiar at the end of testing.

**Figure 7:**
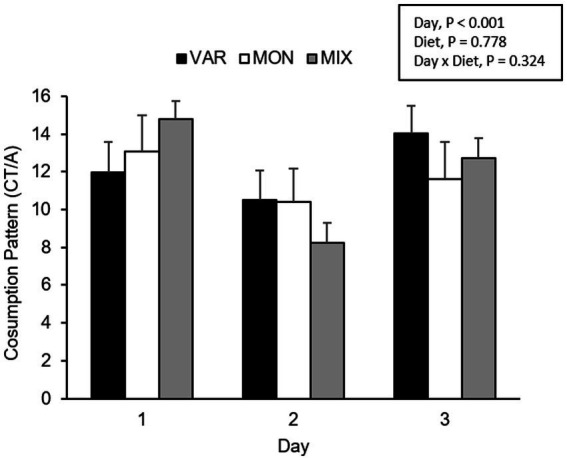
Means (±SEM) of consumption patterns [consumption time (CT)/approaches (A)] of nursery pigs during a consecutive exposure of three feeders (for 30 min each) containing feed with different flavours (orange, chocolate, or grape; VAR), with the same flavour (chocolate; MON) and with a mixture of the three flavours (orange + chocolate + grape; MIX). Results are expressed by pig and experimental day (1, 2, or 3).

**Figure 8:**
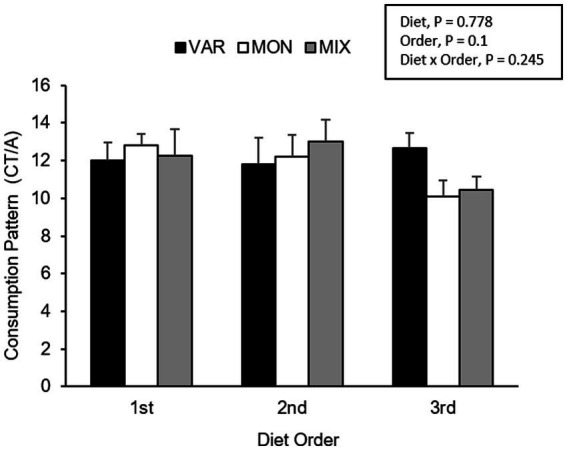
Means (±SEM) of consumption patterns [consumption time (CT)/approaches (A)] (mean ± SEM) of nursery pigs during a consecutive exposure of three feeders (for 30 min each) containing feed with different flavours (orange, chocolate, or grape; VAR), with the same flavour (chocolate; MON) and with a mixture of the three flavours (orange + chocolate + grape; MIX). Results are expressed by pig and diet delivery order (1st, 2nd, or 3rd).

**Figure 9:**
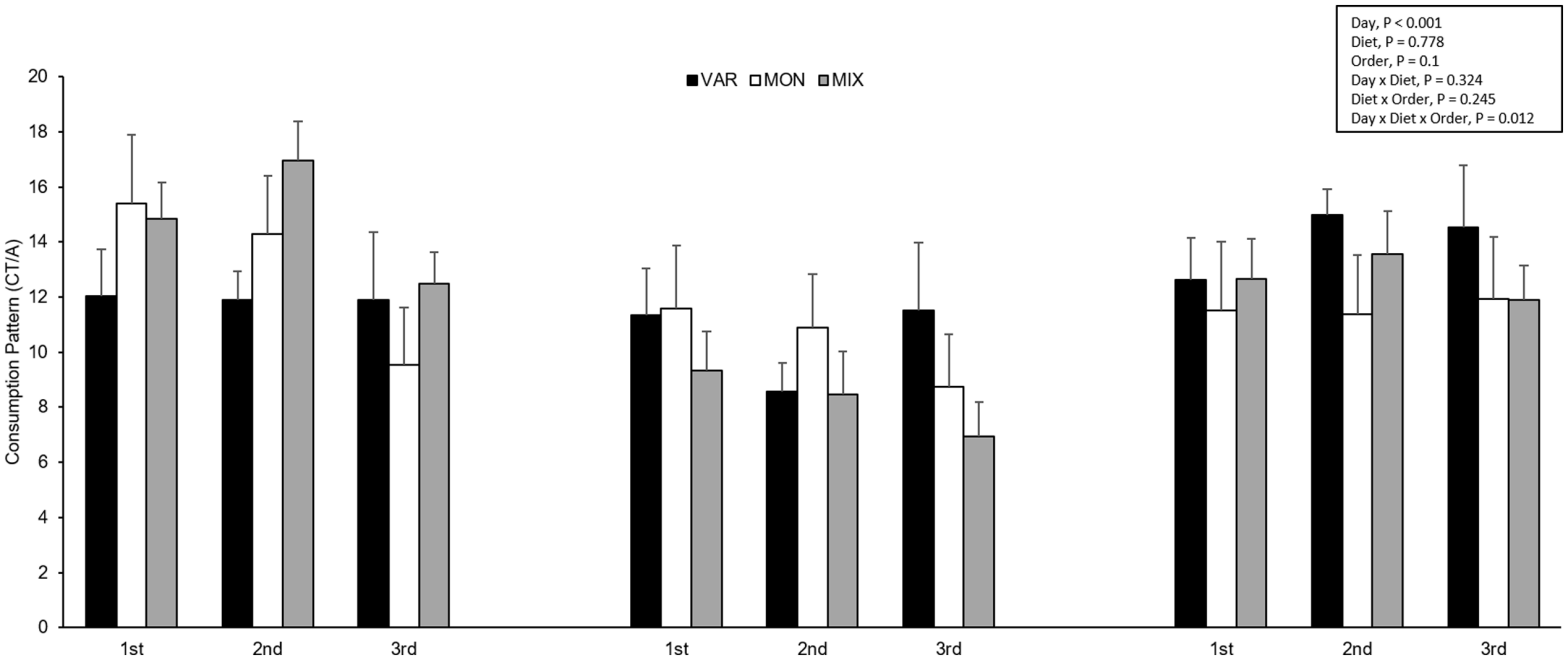
Means (±SEM) of consumption patterns [consumption time (CT)/approaches (A)] (mean ± SEM) of nursery pigs during a consecutive exposure of three feeders (for 30 min each) containing feed with different flavours (orange, chocolate, or grape; VAR), with the same flavour (chocolate; MON) and with a mixture of the three flavours (orange + chocolate + grape; MIX). Results are expressed by pig and diet delivery order (1st, 2nd, or 3rd) and day (1, 2, or 3).

The experimental day influenced the pig’s consumption patterns (*F* 2,15 = 16.29; *p* < 0.001), observing a lower consumption pattern on the second day. No differences between diets were observed in the pig’s consumption patterns (*F* 2,15 = 0.26; *p* = 0.778). The delivery order of feed tended to affect consumption patterns (*F* 2,15 = 2.69; *p* = 0.1), where feed presented the highest hedonic value during its first exposure. The interaction between the treatment and day is presented in Figure 7. Although it is observed that the variety group showed the least consumption pattern on day one and the highest on day 3, the interaction was not significant (*F* 4,15 = 1.27; *p* = 0.324). The interaction between the diet and delivery order is presented in Figure 8. Although this interaction was not significant (*F* 4,15 = 1.52; *p* = 0.245), it is the case that the VAR diet showed the lowest consumption pattern with the first feed delivery and the highest with the last one. Finally, a triple interaction between diet, day and delivery order was observed (*F* 12,15 = 3.52; *p* = 0.012), where the VAR diet presented the lowest consumption pattern during the last delivery on the first day but the highest consumption pattern during the last delivery on the second and the last experimental day (Figure 9): that is, the palatability responses were maintained across the session most clearly in the VAR condition once the flavours were familiar.

## 4. Discussion

Sensory variety could reduce the effect of sensory-specific satiety by increasing the hedonic value of food during animal’s intake (29, 30). However, there is a paucity of information about the effect of flavour variety on the feeding behaviour of nursery pigs. Previous research demonstrated that suckling piglets increased feed exploration and intake when sensory variety was implemented in their diets, by changing multiple sensory properties of the feed, however, no effects on animals’ performance were observed and animals presented no differences in their body weight at weaning (22). Here, we investigated the short-term effect of flavour variety on feed intake and feed palatability in nursery pigs. It was observed that pigs presented an improve in feed intake and perceived palatability when different flavoured feeds were delivered simultaneously or at the end of a consecutive delivery compared with monotonous flavoured diets. A significant interaction between day and diet was found, observing the importance of familiarity of flavours cues to reduce neophobia when sensory variety is implemented to increase voluntary feed intake in nursery pigs. These results could encourage the swine industry to change the way animals are feed, and could improve animal welfare by allowing pigs to express their natural feeding behaviour (20) and increase their perceived palatability (26) by consuming sensory variety diets. Thus, presenting both a challenge and opportunity for the pig industry in terms of animal welfare and sustainability.

### 4.1. Trial 1: simultaneous exposure to flavoured feed

In a natural environment, there are a variety of foods with different nutritional, chemical and physical characteristics available for pigs. These animals are able to select between different consumption options to meet their nutritional requirements even in commercial facilities (20). In Trial 1, no overall differences were observed in pigs’ feed intake when they were offered three pan feeders with different flavoured feeds (VAR) vs. three pan feeders containing the same flavoured feed (MON) or three mixed flavours (MIX) during the test (276 g vs. 234 g vs. 258 g, *p* = 0.276) respectively. However, a clear interaction between the experimental day and treatment was found, whereby the consumption of the variety diet (VAR) increased as the days went on compared to the other diets. It was observed an increase of 64% of feed intake between experimental day 1 and 2 for diet VAR in contrast with only a 7% of increase and a 17% of decrease in feed intake for MON and MIX diets. In agreement with Miller and Holzman (31), it is possible that the animals have experienced fear of consuming different flavours when they were exposed to the sensory cues for the first time. Animals may develop behavioural predispositions oriented to rejecting the consumption of food, whose post-ingestive consequences are unknown, thus avoiding possible toxic effects (32, 33). Pigs without previous experience with particular feeds and its related flavours may display neophobia resulting in a higher latency time to approach novel feeds and a decrease in their intake (34). The negative effects of neophobia are greater at weaning or when new ingredients or additives are added to commercial diets (35). In the present experiment, the animals were not previously exposed to the flavours. Therefore, the effect of neophobia could explain the non-significant difference observed in animals´ consumption between treatments at the start of testing. Similar results were reported by Middelkoop et al. (22), where pigs exposed to novel flavours decrease their feed intake during the first exposures. Although feed neophobia causes pigs to eat small amounts of feed, this behaviour can dissipate with repeated exposure to that feed and its related sensory cues. Thus, animals can verify that the consumption of that feed does not cause negative post-ingestive effects (36). Strategies to increase the familiarity of flavours cues has been reported in suckling and nursery pigs. Probably the most practical strategy is to include those flavours into the gestational diets of sows and prenatally expose pigs to them, generating benefits because of familiarity and associative learning between flavours and the positive effects of aminiotic fluid (35, 37). Another option is to include those flavours in high digestive and palatable diets at the beginning of solid feed consumption.

### 4.2. Trial 2: consecutive exposure to flavoured feed

In addition to the effects of neophobia, a different intake of the flavoured feed was observed related to delivering order (i.e., 1st, 2nd or 3rd delivered pan feeder). In this experiment, pigs’ feed intake decreased considerably in the second pan feeder delivered, and a little more in the third pan feeder delivered (thus, intake was reduced across the session overall). These intake differences were more pronounced in MON treatments, observing an interaction between delivery order and treatment. This lower feed intake as feed exposure increases could be considered a direct consequence of the SSS (9). In the VAR diet, the feed intake was the one that decreased the least, compared with MON and MIX diets. As with Trial 1, this effect was most apparent in the later testing days once the flavours were familiar which decreases neophobia. These results suggest that flavour diversity modifies feed intake in pigs and that they prefer varied diets instead of consuming a diet with similar sensory cues to the one that they experienced before (22). Our findings are in concordance with studies carried out in humans, where the access to a varied diet increases food intake compared to a monotonous one (38–41).

In addition to changes observed in feed intake, the SSS also could affect the pleasure perception, as was observed in the significant effect of delivery order, where the consumption pattern of flavoured feed decreased as the delivery order progressed. This has been previously seen as an effect of repetitive exposure to food in humans (42). The results are consistent with the investigation of the mechanism of SSS, where pleasure perception decreases until consumption stops and, thus, concludes an eating episode (43). Moreover, consumption patterns in VAR treatment were highest in the last feed delivery than in the first one, unlike MON and MIX treatments where the consumption pattern was lower in the last delivery than in the first one. However, only a tendency was observed in the interaction between treatment and delivery order. Results obtained in the VAR treatment show that the effect of SSS was reduced due to sensory changes that produced the delivery of different flavours (8).

Considering the results in Trial 1, an interaction between treatment and day was expected because of neophobia and flavour variety. However, no effects of this interaction were observed on feed intake or consumption patterns. In line with previous studies, where consumption increases when several feed options have been offered throughout the days (44), and similar to the results in Trial 1 where flavours were simultaneously exposed, VAR treatment presented the highest feed intake and consumption pattern on the last experimental day compared to MON and MIX treatments, but the lowest intake and consumption patterns on the first experimental day. Moreover, it is observed that in the third pan feeder delivered, animals had a higher satiety due to continuous exposure to feed. However, VAR treatment in the last pan-feeder delivered presented a smaller decrease in consumption and a higher consumption pattern than MON and MIX compared to the first delivery order.

A triple interaction was observed between treatment, day, and delivery order on feed intake and consumption patterns, where the VAR treatment showed the highest consumption patterns and feed intake on the last day and last delivery order, differing from MON and MIX treatments, which presented lower feed intake and consumption patterns. This could be explained by the neophobia effects on the first day. As the days go by, there is greater exposure to the VAR treatment; the feed became more familiar and consequently, the order effect is higher on the last day. Therefore, the VAR treatment had a better response when satiety occurs during continuous exposure to flavoured feeds, but only when the pigs had a previous experience with those flavours, avoiding neophobia effects.

It appears that if Trial 1, where the feeding options were delivered at the same time, had lasted only 30 min, the interaction between treatment and day would not have been observed. This result contrast with previous research by Ackroff et al. (45) in rats, where no differences were found in solution intake when bottles of sucrose solution with different flavours were offered simultaneously, compared to unflavoured sucrose solutions. Rolls et al. (6) observed that offering a variety of foods to rats successively did not have the same significant positive effects as simultaneous exposure to a variety of food. Nevertheless, this could be explained by the low frequency of the food’s rotation (12 h intervals) on successive exposition. Furthermore, a varied diet treatment has a better response in the SSS when animals are exposed to different food for less than 2 h (39).

Flavours are usually used in the pig industry to enhance feed intake because of their palatability (22) and their sensory continuity effect when milky flavours are incorporated after weaning (25, 46). The present results demonstrated that the variety of flavours, between or within consumption episodes, improved feed intake and palatability in nursery pigs. However, neophobia should be considered (35) when flavours are included for the first time. By repeating the rotation of flavours, we could take advantage of both variety and familiarity. In the present study, flavours were used to generate feed variety since they are easily detected by pigs due to their developed oro-nasal system and because the nutritional content of the diets does not change. However, other sensory stimuli may be used to generate sensory variety in the feed. In humans, it has been shown that presenting the same food in a second dish with different condiments could restore the hedonic value of foods (38). Moreover, SSS can even occur in a simulated feeding where participants chew food but do not swallow it (47). Moreover, it has been shown in humans that the colour and shape of food also have affect SSS (42). Therefore, the SSS is specific to the sensory modality (48). In pigs, studies have shown that feeds that are more diverse in terms of sensory properties increase feed intake (22). Considering this, it is possible that not only flavour could produce effects on the SSS of pigs, but also taste, texture, or colour. It would be important to identify which sensory modality is the most effective in avoiding the effects of SSS.

Dietary variety studies in pigs conducted by Middelkoop et al. (22) have focused on the suckling period, where an improvement in animal welfare but not in the performance of suckling piglets has been reported. Specifically, the animals had an increase in exploratory behaviour, but not in growth performance (22). This last may be due to the number of non-controlled factors during this productive period, such as the presence of the mother and the choice of consumption between milk and feed. Other studies carried out during the rearing period with lambs (49), showed that animals exposed to a multi-forage diet had higher performance (e.g., greater daily gain and dry matter intake) and better welfare parameters (e.g., fewer stereotyped behaviours) compared to animals exposed to single forage. These simple and innovative feeding strategies could be replicated in weaning or fattening pigs. In both productive stages, the feed provided to pigs is often solid and invariant from a point of view of its sensorial properties, generating SSS with its potential negative consequences on performance and welfare. Therefore, a varied diet, that could be rotated weekly or when diet formulation change according to productive stages, could have a positive impact, considering that in the present study there were positive results in terms of palatability and feed intake. Moreover, having a variety of flavours pigs can express their exploratory behaviour at the time of feed consumption. However, it is necessary to complement with behavioural and/or physiological indicators, to determine whether effectively there is an increase in animal welfare, for instance, through the expression of positive affective states by varying the sensory properties of the feed.

## 5. Conclusion

The variety of flavours, between or within consumption episodes may improve feed intake and palatability in nursery pigs. However, is important to consider the effect of neophobia when pigs are exposed to a novel flavour to prevent a possible decrease in their feed intake. The results of this study suggest that sensory varied diets might be used as a strategy to reduce SSS in nursery pigs in conventional industry. Future research must be done to investigate whether a periodic rotation (weekly or when formulation is changed) of feeds that differ in sensory proprieties could be a practical management for pig’s industry to try to increase intake and performance during growing (nursery and/or fattening periods) as has been found in other production systems. Moreover, the increase in perceived feed palatability could improve animal welfare since pigs would increase their pleasure perception for feed when have the opportunity to “choose” (simultaneous exposure) or to received (continuously exposure) different flavoured cues, expressing, somehow, their natural feeding behaviours. Finally, that variety of other sensory properties like taste, texture or colour on sensory specific satiety could be explored in growing animals in order to see the most effective way to reduce the negative effects of sensory monotony in pigs.

## Data availability statement

The raw data supporting the conclusions of this article will be made available by the authors, without undue reservation.

## Ethics statement

All experimental procedures were approved by the Ethical Committee on Animal Experimentation of PUC (No. 190531007). The study was conducted in accordance with the local legislation and institutional requirements.

## Author contributions

JF and DD: conceptualization. JF and LS: methodology, investigation, and supervision. JF, LS, DF, and MV: data curation. JF, EH, and MV: formal analysis. JF: funding acquisition, methodology, project administration, resources, and visualization. EH and JF: writing original draft. JF, DL, and DD: writing review and editing. All authors contributed to the article and approved the submitted version.

## Funding

This research was funded by the National Research and Development Agency (ANID) through the Programme FONDECYT Regular grant number 1200643.

## Conflict of interest

The authors declare that the research was conducted in the absence of any commercial or financial relationships that could be construed as a potential conflict of interest.

## Publisher’s note

All claims expressed in this article are solely those of the authors and do not necessarily represent those of their affiliated organizations, or those of the publisher, the editors and the reviewers. Any product that may be evaluated in this article, or claim that may be made by its manufacturer, is not guaranteed or endorsed by the publisher.
